# Paternalistic Leadership and Subordinates’ Trust in Supervisors: Mediating Effects of Basic Psychological Needs Satisfaction

**DOI:** 10.3389/fpsyg.2021.722620

**Published:** 2021-08-12

**Authors:** Runjia Tang, Yonghong Cai, Heyu Zhang

**Affiliations:** Faculty of Education, Beijing Normal University, Beijing, China

**Keywords:** paternalistic leadership, trust in supervisor, motivation, basic psychological needs, self-determination theory

## Abstract

Subordinates’ trust is critical for a supervisor’s exercise of leadership to effectively influence subordinates’ work outcomes. However, the optimal approach for facilitating trust is still under debate, between instrumentality-based and motivation-based perspectives. On the basis of self-determination theory (SDT), the current study explored the direct effects of paternalistic leadership on trust in supervisors (TS) and the mediating role of the satisfaction of subordinates’ basic psychological needs. In a survey of 1,076 teachers in China, we found that paternalistic leadership affected trust directly, and that subordinates’ need for competence, autonomy, and relatedness also mediated the leadership–trust relationship to different degrees. The theoretical and practical implications of these findings are discussed.

## Introduction

Trust has been increasingly viewed as a central factor determining the effectiveness and efficiency of leadership and organizational management over the last several decades (van der Werff et al., 2019; Legood et al., 2021). Trusting relationships can positively predict desired employee outcomes, including employees’ job satisfaction, effort, performance, and citizenship behavior, and mitigate negative outcomes including employees’ intention to quit (Fulmer and Gelfand, 2012). Therefore, determining how supervisors can promote subordinates’ sense of trust is considered increasingly critical in organizations (van der Werff et al., 2019). Studies have reported findings regarding the antecedents and consequences of trust, among which a variety of leadership styles have been found to be effective for enhancing subordinates’ trust in supervisors (TS) (Fulmer and Gelfand, 2012; Chen et al., 2014; Legood et al., 2021).

Despite the attention trust has received in recent organizational research, current understandings of trust are still under debate. Trust is viewed as a person’s willingness to be vulnerable to the actions of another party based on the expectations of the other party (Mayer et al., 1995). Such conception of trust recognizes that trust encompasses two important components: willingness to be vulnerable, and positive expectations. Scholars have suggested two approaches for analyzing trust (Kramer, 1999). One approach focuses on vulnerability, and views trust as a psychological state involving affective and motivational components. Drawing from an economic perspective, the other approach attaches more importance to the role of expectations and considers trust as a choice behavior based on rationality and efficiency. Furthermore, research studies that combine both approaches have suggested that trust may develop over time from being calculus-based to having relational aspects (Rousseau et al., 1998). In the current study, we define trust as positive expectations and confident decisions that one’s well-being will not be harmed by another party based on an ongoing psychological process.

Based on different understandings of the nature of trust, various theories have been used to analyze the antecedents of trust, among which social exchange theory has been the dominant paradigm in trust research to date (Nienaber et al., 2014). Social exchange theorists emphasize the significance of reciprocity norms, through which a person is motivated to help another party if that other party treats them with genuine care and assistance (Blau, 1964). Although social exchange theory transcends traditional conceptual stress on economic resources by economists and considers social resources as core components, the motivational mechanisms underlying social exchange theory are still based on human rationality. Hence, social exchange theory reflects an instrumental orientation toward human motivation, which suggests that reinforcement processes are critical, and the search for motivation is shifted to an exploration of contextual contingencies that best strengthen such reinforcement during resource exchange processes (Deci and Ryan, 2014; Ryan, 2019). Previous social-exchange-theory-based studies on trust have proposed direct relationships between various contextual factors and trust, while few studies have attempted to further investigate the mediating processes behind decisions about trust (e.g., Wu, 2012; Chen et al., 2014). However, empirical studies in recent years have shown that traditional instrumentality-based trust models have difficulties explaining people’s trust decisions that seem to be irrational (e.g., Baer et al., 2018). Hence, researchers have identified a need for more detailed insight regarding the psychological mechanisms of human trust (van der Werff et al., 2019).

van der Werff et al. (2019) offered a theoretical framework of trust motivation, indicated that human motivation—a central psychological process in understanding the initiation and duration of human behaviors—plays a vital role in trust decisions, and constructed a model of trust motivational processes based on self-determination theory (SDT) to elucidate dynamic intraindividual psychological mechanisms during trusting. SDT assumes that human affect and behaviors are motivated by innate developmental tendencies for growth (Ryan et al., 2019). In motivational processes, people’s psychological needs for competence, autonomy, and relatedness are vital in affecting their psychological wellness and interpersonal relationships with others (Deci and Ryan, 2014). When these needs are satisfied, people feel a strong sense of wellness and a higher propensity toward flourishing, whereas they behave self-protectively when these needs are thwarted (Ryan et al., 2019). In this respect, SDT and the concept of psychological needs offer a useful framework for analyzing how trust (particularly its affective dimension) is facilitated during interactional processes between supervisors and subordinates.

**FIGURE 1:**
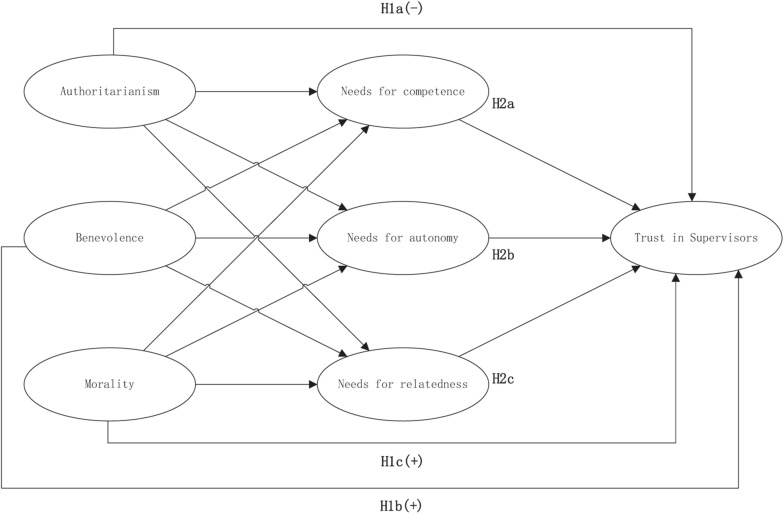
Research model.

In the current study, as shown in Figure 1, we aimed to advance current understandings of trust by incorporating the satisfaction of basic psychological needs as a mediating motivational mechanism in supervisor-subordinate trust relationships, to answer questions about the mechanisms underlying the facilitation of trust. Specifically, we tested how paternalistic leadership, a traditional leadership style that is often employed in East Asian countries, can promote subordinates’ TS directly, and we explored the role that subordinates’ needs for autonomy, competence, and relatedness play in the process. Paternalistic leadership is a particular style of supervisor behavior that shapes the organizational context that affects subordinates’ psychological state and further influences their decisions regarding TS (Pellegrini and Scandura, 2008).

Our study makes two contributions to the trust literature. First and foremost, we extend current research on trust relationships by employing a comprehensive perspective of trust, recognizing the direct effects of leadership on trust while proposing that motivational factors play a key role in the effects of contextual factors (e.g., supervisors’ leadership). Hence, we move beyond the prevailing focus on the direct relationship between trust and its antecedents, and provide a theoretical model including leadership, satisfaction of needs, and trust, which can inform future research and practical leadership behaviors. Second, we specify the concrete dimensions of paternalistic leadership and basic psychological need, depicting a more detailed picture of the relationship among these variables and helping to understand how TS can be facilitated through the specific mechanism that we observe.

### Paternalistic Leadership and Trust in Supervisors

Trust can be influenced by supervisors’ leadership style. Transformational, transactional, authentic, ethical, servant, and empowering leadership have all been found to affect subordinates’ TS to different degrees (Legood et al., 2021). In Eastern Asian countries where collectivistic and Confucian culture is typically valued, paternalistic leadership may play a relatively important role in organizations (Chen et al., 2014). Western theorists often view paternalistic practices in organizations as reflecting supervisors’ requirement for subordinates’ absolute obedience, constituting an entirely negative type of leadership (Weber, 1968). However, Farh and Cheng (2000) have argued that paternalism is not entirely negative, but contains a variety of dimensions, including authoritarianism, benevolence, and morality. Authoritarianism refers to supervisors’ assertion of strong authority and control over subordinates and demands of absolute obedience. Paternalistic leaders typically use rigorous monitoring to convey high expectations of their subordinates and push them to meet performance requirements (Chen et al., 2014). Conversely, benevolence refers to supervisors’ behaviors that show individualized and holistic concern for subordinates’ personal and family well-being. Such care reveals leaders’ respect, support, and willingness to satisfy their subordinates’ diverse needs (Pellegrini and Scandura, 2008). Finally, morality describes supervisors’ unselfish behaviors that demonstrate their personal virtues. To attract subordinates’ respect and identification, paternalistic leaders must show respect for equity and justice, acting as role models (Chen et al., 2014).

Different dimensions of paternalistic leadership have been found to affect subordinates’ TS in various ways (Wu et al., 2012; Chen et al., 2014). Benevolence and morality are positively related to TS, as benevolent leaders’ genuine care and concern strengthen the emotional bond between leaders and followers, and moral leaders gain followers’ trust through the expression of high moral standards and integrity (Wu et al., 2012; Chen et al., 2014). Based on social exchange theory, researchers have proposed that leaders’ supportive treatment of subordinates may enhance subordinates’ sense of obligation to their leaders, resulting in a sense of indebtedness, further facilitating subordinates’ sense of identification with leaders (Rhoades and Eisenberger, 2002; Chen et al., 2014). Research also suggests that benevolence and integrity are critical components of trustworthiness (Mayer et al., 1995). Hence, benevolence and morality are considered to have a direct positive effect on subordinates’ TS. Conversely, authoritarianism negatively impacts subordinates’ trust. Authoritarian leaders’ rigorous monitoring and controlling behaviors typically signal leaders’ distrust in followers’ willingness and capabilities for professional development and independent improvement of their performance (Chen et al., 2014; Mishra and Ghosh, 2020). These unfavorable exchanges can result in followers’ distrust in leaders. Consequently, in the current study, we tested the following hypothesis:

Hypothesis 1: The supervisor’s authoritarianism (H1a) negatively predicts subordinates’ TS, and the supervisor’s benevolence (H1b) and morality (H1c) positively predict subordinates’ TS.

### Self-Determination Theory and the Satisfaction of Basic Psychological Needs

Self-determination theory has been widely used to analyze the motivational mechanisms underlying the relationship between various organizational contexts and employers’ performance and well-being (Ryan et al., 2019). In contrast to the instrumental perspective, SDT assumes an active human nature by which every human being has a universal tendency of movement toward growth, coherence, and wellness (Deci et al., 2017). Furthermore, although people are proposed to have an inherent propensity to develop toward greater growth and integrity, they will not automatically behave in such ways. Contextual factors are thought to have significant effects on human motivation. Specifically, SDT suggests that satisfaction of basic human psychological needs is a critical psychological mediating mechanism between contextual factors and individual affect and behavior (Ryan et al., 2019).

Needs describe organismic necessities for health (Ryan et al., 2019). The concept of human needs finds its root in Maslow’s (1943) need hierarchy theory, which classifies needs into five sequential categories: physiological needs, safety needs, social needs, esteem needs and self-actualization needs. SDT has further specified three types of psychological needs, including the needs for competence, autonomy, and relatedness, as essential nutrients for high autonomous motivation (Deci et al., 2017). The need for competence (NC) refers to a person’s feeling of being effective in their interactions with the environment and the experience of having opportunities to express and practice their capacities. The need for autonomy (NA) refers to a person’s feeling of choice and concurrence with their own actions. The need for relatedness (NR) refers to the need for a feeling of belonging and connection with others and a sense of being cared for by others and caring for those others (Ryan et al., 2019). Need hierarchy theory focuses on the extent to which people’s diverse needs are satisfied and suggests that needs at higher levels can be sensitized only when the lower needs are satisfied. However, SDT also places a strong emphasis on how contextual factors can either satisfy or thwart people’s basic psychological needs and proposes that the three basic needs are universal and contribute to people’s well-being only when they are all fulfilled (Mishra and Ghosh, 2020).

The use of SDT to analyze how human motivation functions in interpersonal relationships are rooted in research on romantic relationships (van der Werff et al., 2019). Self-determination theorists have developed relationship motivation theory (RMT), with the central proposition that people have a fundamental motive to feel meaningfully connected with others and the satisfaction of all three basic psychological needs jointly contributes to flourishing healthy interpersonal relationships (Deci and Ryan, 2014; Ryan et al., 2019). A trusting relationship yields a true sense of meaningful connection, through which feel they are genuinely cared for and acknowledged (McAllister, 1995). By contrast, in relationships that are based on instrumental utilization of others, people will not experience a sense of belongingness but are more likely to feel frustrated in the satisfaction of connection. According to SDT, NR represents a natural driving force for interpersonal relationships because relatedness is viewed as not merely important, but essential to human wellness (Deci and Ryan, 2014). Even people who actively disconnect from others will nonetheless suffer psychological illness due to the lack of fulfillment of relatedness. SDT and RMT differs from other theoretical explanations of close interpersonal relationship because the satisfaction of basic needs represents people’s intrinsic nature to join groups that physically and emotionally connect them with others. Conversely, some perspectives generate from instrumental views, such as physical security or resource exchanges, can only explain people’s extrinsic motivation, which may play a relatively smaller role in trust because trust is genuine recognition and acknowledgment and cannot be gained by instrumental interpersonal relationships. In addition to NR, SDT posits that NC and NA could also facilitate positive relationship outcomes (Deci and Ryan, 2014). On the one hand, mutually positive feedback and a sense of effectiveness are important for sustaining high-quality trusting relationships. On the other hand, trust is not a relationship involving manipulation or power differences, but rather a balanced affective and emotional connectedness involving mutuality and respect. Thus, a sense of autonomy and choice is vital in trusting relationships. Overall, basic psychological needs reflect people’s psychological processes during interactions with others, and function as critical motivational mechanisms through which contextual factors influence decisions regarding trust.

### Paternalistic Leadership, Needs Satisfaction, and Trust in Supervisors

The three dimensions of paternalistic leadership may have different effects on subordinates’ basic psychological needs (Pellegrini and Scandura, 2008; Chen et al., 2014). First, authoritarianism tends to negatively affect subordinates’ three needs. Although authoritarian leaders have high expectations of subordinates’ competency and performance, rigorous monitoring and controlling approaches may lead to feelings of pressure and threat among subordinates, which could reduce the satisfaction of their NC and NA. Further, hierarchical relationships may signal subordinates’ inferior roles, leading to non-fulfillment of NR (Pellegrini and Scandura, 2008). A study in India reports that strict and unconstructive monitoring frustrates employees’ satisfaction of basic needs (Mishra and Ghosh, 2020). Other empirical studies also indicate that subordinates’ basic psychological needs will be much less satisfied when leaders put heavy pressure on them (van den Broeck et al., 2016). Conversely, if leaders are able to create an organizational climate which celebrates autonomy, subordinates’ satisfaction of basic needs will be significantly facilitated (Balaguer et al., 2012; Yu et al., 2015). Second, benevolence and morality are likely to promote satisfaction of subordinates’ psychological needs. Benevolent leaders show respect, care, and support for subordinates through individualized consideration and efforts to satisfy subordinates’ feelings and needs. Such benevolent leadership is expressed not only in subordinates’ individual work, but also in their daily life, and extends to their family and friends (Chen et al., 2014). Additionally, benevolent leaders offer timely and appropriate assistance and allocate substantial energy and resources to supporting subordinates’ professional development (Pellegrini and Scandura, 2008). Hence, benevolence may facilitate fulfillment of subordinates’ NC and NR. However, benevolence does not involve equal treatment or equivalent status between the supervisor and subordinates. Rather, the leader uses benevolent attitudes and behaviors toward subordinates with an underlying signal of power distance (Chen et al., 2014). Thus, whether benevolent leadership facilitates subordinates’ satisfaction of NA is unclear. Overall, benevolent leaders often provide ample and helpful resources to subordinates, and studies have shown that people’s satisfaction of all three needs will increase when there are abundant work resources (van den Broeck et al., 2016). Moral leaders show a high level of virtue (e.g., equity, justice, and integrity). By exhibiting unselfish behaviors, moral leaders act as role models and gain affective commitment from subordinates (Pellegrini and Scandura, 2008). When leaders demonstrate that they will not abuse power for their own benefit, subordinates tend to be more willing to express their ideas freely and actively strive to solve the problems faced in their work, enhancing their professional abilities and the satisfaction of NC (Chen et al., 2014). However, unlike ethical leadership, moral leadership does not involve subordinates’ participation in decision-making (Chen et al., 2014), and thus its relationship with the NA requires further exploration.

Previous studies have shown that leaders’ needs-supportive behaviors can positively affect employees’ needs satisfaction, and lead to increased TS (Deci et al., 1989). La Guardia et al. (2000) reported that when students’ three basic needs were fulfilled, they felt a higher level of security in their attachments with others, were more satisfied with their interpersonal relationships and were more willing to rely on their partners. A series of studies conducted by Patrick et al. (2007) confirmed that satisfaction of each need positively predicted individuals’ well-being, relationship quality, and effectiveness in managing conflict within interpersonal relationships. Other empirical studies have also demonstrated that when people’s three basic needs are satisfied, they are more likely to develop a good interpersonal relationship with others and gain a higher level of subject well-being (Eakman, 2014; Chen et al., 2015). Taking these previous findings together, we hypothesized that:

Hypothesis 2: The satisfaction of subordinates’ NC, NA, and NR will mediate between authoritarianism (H2a), benevolence (H2b), morality (H2c) and TS.

## Materials and Methods

### Participants

Our study collected data from a survey of 36 primary and secondary schools (18 primary and 18 secondary schools) in northern China. A stratified cluster sampling method was employed. First, we randomly selected the sample schools in the research region. Second, a questionnaire survey was delivered to all teachers in the selected schools. A total of 1,308 questionnaires was distributed, and 1,076 valid questionnaires were obtained (effective recovery rate = 82.26%).

Among the valid responses, there were 155 male teachers (14.4%) and 921 female teachers (85.6%). The surveyed teachers were 35.53 years old on average (SD = 7.91) and had an average of 13.32 years of teaching experience (SD = 8.96). Regarding educational background, 181 teachers had a high school degree or associate degree (16.8%), 852 had a bachelor’s degree (79.2%), and 43 had a master’s or doctoral degree (4.0%).

### Measures

All variables in the research model were measured using five-point Likert-type scales, ranging from “1 = strongly disagree” to “5 = strongly agree.” Two research participants who are proficient in English worked separately and translated the original scales into Chinese. After translation, these two translators worked together and came up with an agreed version of translation. Prior to our survey, 30 primary and secondary school teachers were interviewed to share how they interpreted paternalistic leadership, basic psychological need, and trust. We gave the translated scales to the teachers, and they were asked to judge whether the questions could accurately express what we intended to measure, and if not, what confused them. We kept the questions that could be accurately understood by teachers unchanged, and discussed teachers further on the questions that they had difficulty in figuring out the meaning. These confusing questions were then adapted into a language that school teachers often used in school life, making it easier for teachers to understand. For example, Mascall et al. (2009) offer a question on TS that originally put as “I feel a strong loyalty to our school leaders,” and we found that it was hard for school teachers in China to connect these words to the concept of trust in their school leaders. Hence, this question was revised as “I always support my school leader’s daily work.”

### Paternalistic Leadership: Authoritarianism, Benevolence, and Morality

Authoritarianism, benevolence, and morality were measured based on the adaptation of a widely used paternalistic leadership scale developed by Cheng et al. (2004). There are four items regarding authoritarianism (Cronbach’s α = 0.86; a sample item is, “Almost all decisions in my school are determined by my school supervisor.”), three items regarding benevolence (Cronbach’s α = 0.88; a sample item is, “Beyond school affairs, my supervisor often expresses concern about my daily life.”), and four items regarding morality (Cronbach’s α = 0.91; a sample item is, “My school supervisor won’t use his/her authority to seek special privileges for himself/herself.”). Taking three subscales as a whole, confirmatory factor analysis (CFA) indicated a good model fit (χ^2^ = 293.5; df = 41; χ^2^/df = 7.16; GFI = 0.95; CFI = 0.97; TLI = 0.96; RMSEA = 0.076).

### Satisfaction of Basic Psychological Needs: Need for Competence, Autonomy, and Relatedness

We adapted the Basic Need Satisfaction at Work Scale from Deci et al. (2001). There were three items on each dimension. For NC, Cronbach’s α = 0.80; a sample item was “My supervisor and colleagues often tell me that I am good at teaching”; for NA, Cronbach’s α = 0.62; a sample item was “I can freely express my opinions when I try new teaching initiatives,” and for NR, Cronbach’s α = 0.73; a sample item was “I get on well with my supervisor, colleagues, and students.” CFA indicated a good model fit (χ^2^ = 89.3; df = 24; χ^2^/df = 3.72; GFI = 0.98; CFI = 0.97; TLI = 0.96; RMSEA = 0.050).

### Teachers’ Trust in Supervisors

We measured teachers’ TS using an adapted version of the teacher trust in school leaders scale of Mascall et al. (2009). An inspection of modification indices indicated that residuals of two observed items correlated with each other. After correlating their residuals, we obtained a good model fit (χ^2^ = 4.53; df = 1; χ^2^/df = 4.53; GFI = 0.99; CFI = 0.99; TLI = 0.99; RMSEA = 0.057).

### Data Analysis

We used SPSS 24.0 to test the correlation of the variables, and used AMOS 21.0 to conduct CFA, test the structural equation model (SEM), and calculate specific mediating effects with a bootstrapping method (MacKinnon et al., 2002). SEM is a useful statistical method to test the hypothesized relationships among research variables, especially for latent variables (Byrne, 2001). If the hypothesized model fits the data well, the proposed relationships among variables will be statistically supported (Byrne, 2001). To further explore the mediating role played by NA, NC, and NR, a calculation of specific mediating effects was also conducted. In both the SEM analysis and the calculation of mediating effects, a bootstrapping method with a sample of 2,000 was employed to obtain the 95% bias-corrected confidence intervals, which offers a more accurate result for the analysis (MacKinnon et al., 2002).

## Results

### Discriminant Validity and Common Method Variance Analysis

In order to test the discriminant validity of our measures, the goodness-of-fit of models ranging from seven-factor to single-factor was examined. The results in Table 1 indicate that the seven-factor model fits the data best, suggesting that the three dimensions of paternalistic leadership, the satisfaction of NC, NA, and NR, and teachers’ trust in their supervisors are seven distinct variables, and thus the discriminant validity of the measures is confirmed.

**TABLE 1:** The results of discriminant validity test of the relationships among all variables.

No.	Model	χ ^2^	Df	χ ^2^/df	CFI	GFI	RMSEA	SRMR
M1	7-factor (Au; Be; Mo; NC; NA; NR; TS)	818.55	231	3.54	0.96	0.94	0.049	0.048
M2	6-factor (Au; Be + Mo; NC; NA; NR; TS)	1,324.00	237	5.59	0.92	0.89	0.065	0.054
M3	6-factor (Au; Be; Mo; NC + NA; NR; TS)	1,054.47	237	4.45	0.94	0.92	0.057	0.062
M4	5-factor (Au + Be + Mo; NC; NA; NR; TS)	3,520.03	242	14.55	0.77	0.74	0.112	0.102
M5	5-factor (Au; Be; Mo; NC + NA + NR; TS)	1,210.86	242	5.00	0.93	0.91	0.061	0.065
M6	4-factor (Au + Be + Mo + TS; NC; NA; NR)	4,513.42	246	18.35	0.70	0.69	0.127	0.108
M7	4-factor (Au; Be; Mo; NC + NA + NR + TS)	2,521.52	246	10.25	0.84	0.79	0.093	0.090
M8	3-factor (Au + Be + Mo; NC + NA + NR; TS)	3,814.57	249	15.32	0.75	0.72	0.115	0.107
M9	2-factor (Au + Be + Mo + TS; NC + NA + NR)	4,799.01	251	19.12	0.68	0.67	0.130	0.113
M10	2-factor (Au + Be + Mo; NC + NA + NR + TS)	5,121.68	251	20.41	0.65	0.65	0.134	0.124
M11	1-factor (Au + Be + Mo + NC + NA + NR + TS)	6,284.81	253	24.84	0.57	0.60	0.149	0.137

*Au, authoritarianism; Be, benevolence; Mo, morality; NC, need for competence; NA, need for autonomy; NR, need for relatedness; TS, trust in supervisors.*

Because all of the data were collected from the same group of teachers at the same time, there was possibility for common method variance (CMV) that might affect the results (Podsakoff et al., 2012). Harman’s single-factor CFA, wherein all variables were created as a single construct, was used to examine CMV (Podsakoff et al., 2003). The logic underlying this test is that if CMV is a significant problem, the results of single-factor CFA should fit the data. Table 1 (M11) shows that the single-factor model fits the data very poor (χ^2^ = 6284.81, df = 253, χ^2^/df = 24.84, GFI = 0.60, CFI = 0.57, RMSEA = 0.149, SRMR = 0.137). Further, Podsakoff et al. (2003) suggested that Harman’s single-factor CFA is likely to provide reassurance in most circumstances, and thus cannot guarantee the non-existence of CMV. Therefore, a further test was used in which a latent factor was included in the model. Compared with the goodness-of-fit of the original theoretical model (M1 in Table 1, CFI = 0.958), the new model showed a small increase (CFI_new_ = 0.965, ΔCFI = 0.011), which is below the 0.05 threshold (Little, 1997). The results of these tests indicated that there was no significant evidence of CMV in the data used.

### Descriptive Statistics: Means, Standard Deviations, and Correlations

The reliability of measurements, means, standard deviations and correlations between variables are shown in Table 2. Among all independent variables, authoritarianism was significantly negatively related to NC (*r* = −0.08, *p* < 0.01), NR (*r* = −0.11, *p* < 0.01), and TS (*r* = −0.14, *p* < 0.01). However, it had a significant positive correlation with NA (*r* = 0.25, *p* < 0.01), suggesting the opposite relationship to our hypothesis 2. Additionally, benevolence and morality showed significant positive relationships with all hypothesized mediating variables and dependent variables, supporting our hypothesis 1 and 2.

**TABLE 2:** Means, standard deviations, correlations, and scale reliability.

	M	SD	1	2	3	4	5	6	7	8
1 Au	2.94	1.00	(0.86)							
2 Be	3.65	0.95	0.03	(0.88)						
3 Mo	3.95	0.87	−0.13**	0.76**	(0.91)					
4 NC	4.12	0.68	−0.08*	0.20**	0.28**	(0.80)				
5 NA	3.31	0.69	0.25**	0.20**	0.13**	0.18**	(0.62)			
6 NR	4.14	0.62	−0.11**	0.31**	0.33**	0.57**	0.20**	(0.73)		
7 BPN	3.86	0.49	0.04	0.32**	0.33**	0.79**	0.64**	0.78**	(0.81)	
8 TS	3.80	0.87	−0.14**	0.61**	0.66**	0.32**	0.15**	0.38**	0.38**	(0.88)

Au, authoritarianism; Be, benevolence; Mo, morality; NC, need for competence; NA, need for autonomy; NR, need for relatedness; BPN, basic psychological need; TS, trust in supervisors. Internal reliability values for the constructs are shown in parentheses on the diagonal; **p < 0.01 and *p < 0.05.

### Paternalistic Leadership and Trust in Supervisor

Using SEM, stepwise tests and specified mediating effect tests were employed to examine each of our research hypotheses. In the first step, only authoritarianism, benevolence, morality, and TS were included in Model 1, and a good model fitness was acquired (χ^2^ = 469.8; df = 84; χ^2^/df = 5.59; GFI = 0.95; CFI = 0.97; TLI = 0.96; RMSEA = 0.065). Supporting H1a, H1b, and H1c, the results of SEM (M1 in Table 3) indicated that all three types of leadership were significantly related to TS. Specifically, as hypothesized, authoritarianism negatively predicted TS (β = −0.11, *p* < 0.01), and benevolence (β = 0.28, *p* < 0.01), and morality (β = 0.47, *p* < 0.01) predicted TS in a positive way.

**TABLE 3:** Standardized results of stepwise mediation test of basic psychological need.

	M1	M2			
	
	TS	NC	NA	NR	TS
Authoritarianism	−0.11**	−0.04	0.33**	−0.13*	−0.13**
Benevolence	0.28**	−0.08	0.32*	0.16	0.22**
Morality	0.47**	0.39**	0.03	0.28*	0.42**
NC					0.08*
NA					0.14**
NR					0.11**
R^2^	0.55	0.11	0.22	0.21	0.59

TS, trust in supervisors; NC, need for competence; NA, need for autonomy; NR, need for relatedness. **p < 0.01 and *p < 0.05.

### The Mediating Effect of the Need for Competence, Autonomy, and Relatedness

**FIGURE 2:**
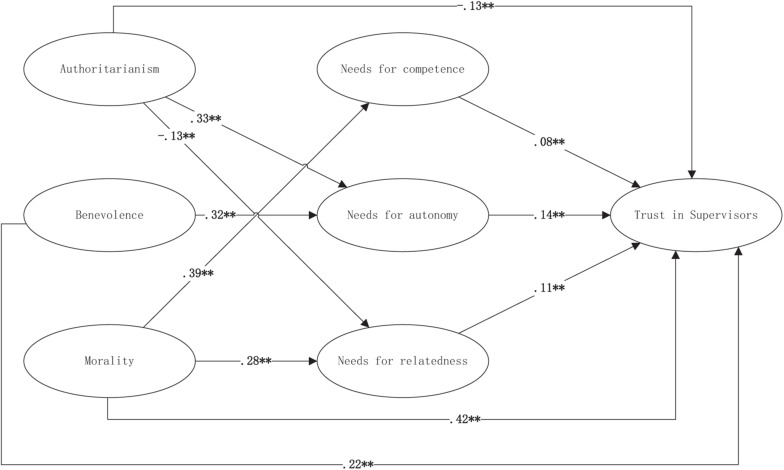
Standardized results of the research model. Non-significant paths are not shown in this figure; ^∗^*p* < 0.05 and ^∗∗^*p* < 0.01.

In the second step, three mediating variables, including teachers’ satisfaction of NC, NA, and NR were added to Model 2. The model also exhibited a good fit (χ^2^ = 1,213.0; df = 234; χ^2^/df = 5.18; GFI = 0.92; CFI = 0.93; TLI = 0.92; RMSEA = 0.062). The results (M2 in Table 3 and mediating effects in Table 4) revealed that NA positively mediated between authoritarianism and TS (β_authoritarianism–NA_ = 0.33, *p* < 0.01; β_NA–TS_ = 0.14, *p* < 0.01; Mediating Effect_authoritarianism–NA–TS_ = 0.042, *p* < 0.01) as well as between benevolence and TS (β_benevolence–NA_ = 0.32, *p* < 0.05; β_NA–TS_ = 0.14, *p* < 0.01; Mediating Effect_benevolence–NA–TS_ = 0.043, *p* < 0.05). NC mediated positively between morality and TS (β_morality–NC_ = 0.32, *p* < 0.01; β_NC–TS_ = 0.08, *p* < 0.05; Mediating Effect_morality–NC–TS_ = 0.35, *p* < 0.05). NR exhibited a negative mediating effect between authoritarianism and TS (β_authoritarianism–NR_ = −0.13, *p* < 0.05; β_NR–TS_ = 0.11, *p* < 0.01; Mediating Effect_authoritarianism–NR–TS_ = −0.012, *p* < 0.05), and a positive mediating effect between morality and TS (β_morality–NR_ = 0.28, *p* < 0.05; β_NR–TS_ = 0.11, *p* < 0.01; Mediating Effect_morality–NR–TS_ = 0.034, *p* < 0.05). Moreover, given the significant direct effects of authoritarianism, benevolence, and morality on TS (direct effect in Table 4), the mediations of all three needs were partial. Thus, H2b and H2c were completely supported, whereas H2a was only partially supported due to the positive mediating effect of NA. Figure 2 shows the overall results of our hypothesized model.

**TABLE 4:** Standardized results of direct effects and mediating effects on trust in supervisor.

Parameter estimates	Effect	95% BC lower	95% BC upper	P
**Direct effect**				
Au→TS	–0.133	–0.194	–0.065	0.001
Be→TS	0.222	0.084	0.347	0.005
Mo→TS	0.416	0.314	0.621	0.003
**Mediating effect**				
Au→NC→TS *(H2a)*	–0.003	–0.013	0.004	0.293
Au→NA→TS *(H2a)*	0.042	0.015	0.088	0.003
Au→NR→TS *(H2a)*	–0.012	–0.031	–0.003	0.011
Be→NC→TS *(H2b)*	–0.007	–0.055	0.009	0.309
Be→NA→TS *(H2b)*	0.043	0.006	0.114	0.020
Be→NR→TS *(H2b)*	0.017	–0.010	0.063	0.165
Mo→NC→TS *(H2c)*	0.035	0.006	0.106	0.014
Mo→NA→TS *(H2c)*	0.005	–0.035	0.062	0.687
Mo→NR→TS *(H2c)*	0.034	0.004	0.105	0.024
**Total effect**				
Au→TS	–0.105	–0.164	–0.051	0.003
Be→TS	0.276	0.138	0.399	0.005
Mo→TS	0.481	0.350	0.617	0.001

*Au, authoritarianism; Be, benevolence; Mo, morality; TS, trust in supervisors; NC, need for competence; NA, need for autonomy; NR, need for relatedness.*

## Discussion

The present study investigated the direct relationships between three dimensions of paternalistic leadership (authoritarianism, benevolence, and morality) and teachers’ TS. Further, the mediating effect of basic psychological needs satisfaction (i.e., NC, NA, and NR) was explored.

First, the results of the present study suggested a negative direct relationship between authoritarianism and TS, and positive benevolence-TS and morality-TS direct relationships, which confirms that paternalistic leadership is a multi-dimensional construct, and supports the explanatory power of social exchange theory and other perspectives involving a direct effect of leadership on trust. Weber (1968) views paternalistic leaders as equivalent to autocrats and proposing a negative connection between authoritarianism and desired employee and organization outcomes, and the present study supported the negative aspects of paternalistic leaders’ controlling behaviors. Authoritarianism often triggers negative emotions among subordinates, such as anger and fear, and weakens their positive feelings and subjective well-being (Chen et al., 2014). Furthermore, absolute control over subordinates impedes free and equal exchange between supervisors and followers, leading to subordinates’ feeling of not being adequately recognized or respected (Pellegrini and Scandura, 2008). All these factors resulting from negative interactions are considered core emotional antecedents of a lack of affective trust. However, the benevolent and moral aspects of paternalism appeared to positively predict TS, in accord with findings previously (Cheng et al., 2004). Benevolent leaders typically express genuine and sincere concern about their subordinates’ personal welfare regarding work issues and daily life. These positive exchanges often lead to feelings of obligation or indebtedness among followers, and a sense that they need to repay their leader’s kindness (Legood et al., 2021). As a result, subordinates tend to have more positive expectations of their leaders’ future behaviors. Additionally, subordinates’ positive emotions are likely to be induced by benevolent treatment from leaders, forming an affective foundation for TS (Chen et al., 2014). Similarly, moral leaders exhibit high levels of virtue and behave in accordance with moral standards. Knowing that their supervisor will not take advantage of others for their own benefit, followers tend to show greater affective commitment and loyalty to their supervisor (Chen et al., 2014). The current results revealed that morality had the strongest effect on TS. One potential reason for this result is the direct link between leaders’ morality and the integrity aspect of trustworthiness (Mayer et al., 1995). Subordinates may believe more strongly in their leaders’ behaviors and more confident that it is safe to expose their vulnerability because of their leaders’ high moral standards. Additionally, traditional Chinese culture and Confucianism may also play important roles in the effectiveness of moral leadership. According to Confucianism, the primary goal of administration is to develop subordinates’ morality, educating them as righteous people (Ip, 2009). To achieve this, leaders themselves need to demonstrate high moral standards, and whether their behaviors can match their word is a core criterion of their leadership. Moral leaders are more likely to gain subordinates’ trust and stimulate their sense of responsibility to strive for the wellness of the entire organization and society. Conversely, if leaders often violate moral standards and regulations, subordinates will also tend to abuse their power for their own personal benefit, and a climate of distrust will be generated. In sum, leaders’ morality is a core component of effective leadership and is valued more highly than professional ability in China.

Second, expanding on previous research on the direct relationship between leadership and trust, we further tested the mediating effects of three basic psychological needs. The results largely supported our research hypothesis 2. Specifically, NA mediated both authoritarianism-TS and benevolence-TS, NC only mediated between morality and TS, while NR mediated both authoritarianism-TS and morality-TS. It has previously been argued that basic psychological needs satisfaction is universal for explaining spontaneous human feelings and behaviors (Deci and Ryan, 2008), and the present findings provide further support for this notion. However, satisfaction of NA exhibited a positive mediating effect between authoritarianism and TS, contrary to our hypothesis 2.

The important role of NR in determining interpersonal relationships was confirmed in the current study. SDT suggests that people share a universal inherent need to feel meaningfully connected and related to others (Deci and Ryan, 2014). NR is independent of instrumental desire, such as physical security and economic or social resource exchanges. Thus, relatedness is considered to be essential to human wellness, without which people feel difficulty living a vital and energetic life. Indeed, SDT-based studies have demonstrated that people feel a lower sense of engagement, interest, and trust in interpersonal relationships when they hold an extrinsic orientation to relate to others (Wild et al., 1997). Furthermore, individuals who hold such extrinsic and instrumental perspectives are more likely to connect with a similar extrinsically oriented partner, resulting in each side treating the other as an object instead of a developing organism (Williams et al., 2000). Conversely, healthy interpersonal relationships developed on the basis of the inherent need for relatedness are critical throughout the lifespan, and necessary for psychological development (Deci and Ryan, 2014). The current results revealed that the moral dimension of paternalistic leadership significantly enhanced the satisfaction of teachers’ NR. Rempel et al. (1985) found that subordinates were more intrinsically motivated when their leaders showed a higher level of moral standards. Specifically, when leaders explicitly demonstrated their fairness, unselfishness, and trustworthiness, followers tended to be more willing to develop an emotional bond in addition to an economic relationship with their leaders (Wu et al., 2012). The current finding that authoritarianism was negatively related to NR may be expected because authoritarian leaders’ vigorous demands for the benefit of the organization and the neglect of followers’ desire for communication signal an instrumental aspect of the supervisor-subordinate relationship (Mishra and Ghosh, 2020). Under these circumstances, subordinates follow their leaders’ orders to obtain social or economic resources or avoid punishment, and their NR is likely to be harmed. Thus, in accord with other SDT-based studies, the current findings support the important mediating role that NR plays in the facilitation of trust in organizations.

The current results also confirmed a mediating role of the NA in leadership and TS. In a trusting relationship, a person does not have the intention of controlling the partner, or a desire for one partner to depend on the other. Rather, trusting relationships are close interpersonal relationships involving consent and mutuality (Deci and Ryan, 2014). Within trusting relationships, partners do not exercise authority over one another, but respect and encourage other’s choices. Hence, autonomy is crucial in initiating and sustaining healthy trusting relationships. When individuals perceive a higher level of autonomy from their partners, they report greater relationship satisfaction, attachment security with and emotional reliance on the partners, and they are more likely to include the partner in their own sense of self (Deci et al., 2006). When there are disagreements within the relationship, autonomy-supported partners show less defensiveness and more understanding of other’s opinions (Knee et al., 2005). Similar to the findings regarding relatedness, autonomy-supported individuals tend to be more likely to develop satisfying high-quality relationships, and to show a greater ability to maintain such relationships in the long term compared with individuals with controlling motivations (Blais et al., 1990). In terms of the facilitation of trustees’ NA, the current results revealed that benevolent leadership behaviors had a positive effect. In addition to their individualized concern for subordinates’ work and life well-being, benevolent leaders actively support subordinates who are in trouble, and provide professional developmental opportunities and respect subordinates’ NA during the process of personal growth (Dedahanov et al., 2019). However, our results also suggested a positive impact of authoritarianism on the satisfaction of subordinates’ NA, contrary to our hypothesis and previous findings of negative relationships between these factors (Pellegrini and Scandura, 2008). One possible explanation is that, in SDT, autonomy refers to one’s subjective feeling of psychological freedom when engaging in a behavior (Chong and Gagné, 2019). Thus, autonomy in SDT is a limited subjective freedom. Individuals can theoretically feel autonomously satisfied while having to obey others’ requests. In Chinese primary and high schools, there is a long history of hierarchical administrative structure and power distance between supervisors and teachers. Moreover, although teachers do not have the power to determine school administrative issues, they often have a high degree of freedom in their classrooms. Therefore, the controlling aspects of paternalistic leadership may not depress teachers’ NA in their classrooms. Future studies may further test such relationships in other professional and cultural contexts to obtain more detailed insight.

NC also showed a significant mediating effect between morality and trust. RMT proposes that flourishing interpersonal relationships require the satisfaction of individuals’ three needs (Deci and Ryan, 2014). Like the other two types of need, NC is also a key antecedent influencing individuals’ psychological health and wellness. When NC is thwarted, people show signs of ill-being. A previous study showed that the fulfillment of NC could independently contribute to positive relationship outcomes, such as security of attachment (La Guardia et al., 2000). However, compared with relatedness and autonomy, competence is theorized to have the weakest contribution to the quality of interpersonal relationships because competence is often not a primary goal of trust, friendship, or other relationships (Deci and Ryan, 2014). The results of the current study also revealed that NC had the weakest effect on TS. Furthermore, morality was found to facilitate satisfaction of subordinates’ NC. Moral leaders not only show a high level of virtue, but also strive for organizational goals and to improve their professional capabilities (Dedahanov et al., 2019). Hence, leaders’ morality offers a role model for both moral standards and professional capacities. If subordinates are impressed by their leaders, they are more likely to enhance their ability and increase their competence.

Overall, the current results revealed that paternalistic leadership can affect subordinates’ TS both directly and through the mediation of psychological needs. Moreover, compared with the direct effects, the mediating effects of needs account for a smaller proportion of influence, which indicates that social exchange theory has stronger explanatory power in trust relationships, although SDT can also contribute to the understanding of trust.

### Theoretical Implications

The current study makes several theoretical contributions to existing literature on trust. First, we incorporated a social exchange perspective and trust motivation perspective by building a theoretical model to examine both the direct and indirect effects of paternalistic leadership on trust. Our findings support the effectiveness of social exchange theory in explaining the facilitation of trust. Trust is an expectation of trustees’ future behaviors according to the judgment of trustors’ previous interactions with them (Dirks and Ferrin, 2001). Hence, social exchange processes are an important way for trustors to understand trustees and offer opportunities for trustors to obtain as much information as possible to evaluate the partner’s trustworthiness and decide whether to expose their vulnerability to the partner in future interactions. Positive exchanges will strengthen the individual’s confidence about the partner’s benevolence and integrity, whereas negative exchanges will lead to feelings of fear and discomfort, all of which generate direct facilitating or impeding effects on trust.

On the basis of the trust motivational framework by van der Werff et al. (2019) and the constructs of SDT, we also examined the mediating role of basic psychological needs and found that trust relationships, particularly their affective aspects, are also a psychological state that is influenced by motivational processes. SDT views trust as a part of inherent psychological well-being for all humans (Deci and Ryan, 2014). Thus, like other intrinsically driven affects and behaviors studied in SDT, we propose that the satisfaction of individuals’ NC, NA, and NR forms the motivational mechanisms underlying their sense of trust. The present results showed that leadership exhibits various relationships with different types of need, and different psychological needs also affect trust to various degrees. Moreover, the mediating effect was not complete, and was relatively small compared with the direct effect of leadership. Thus, it should also be noted that only a small component of trust appeared to be self-determined, rather than its entirety. Social exchange theory and other instrumental perspectives may still be useful for understanding the formation of trust in interpersonal relationships.

Overall, our trust model offers a potentially useful theoretical framework for researchers to further explore the facilitation or diminishment of trust in various settings, providing a starting point for future studies to involve a wide range of motivational theories into research on trust.

### Practical Implications

The present study also has several practical implications. Paternalistic leadership is characterized by its multi-dimensionality. Whereas authoritarianism represents the controlling aspect of paternalism and exhibits a negative relationship with trust, benevolence and morality are the encouraging sides of paternalism, and positively affect subordinates’ trust. Therefore, paternalistic leaders should pay close attention to their behaviors in relation to each aspect, which means that the exercise of paternalistic leadership is an art of balance. The benevolent and moral aspects of paternalism are effective for not only promoting employee performance, as many previous studies have suggested (Pellegrini and Scandura, 2008; Chen et al., 2014), but also strengthening its effectiveness by further enhancing the interpersonal relationships between the supervisor and followers. Thus, supervisors should show more benevolence and try to become a moral standard in the organization. Besides, although authoritarianism might contribute to desired organizational outcomes in other important ways, leaders should not abuse their authority, as these controlling approaches may generate other undesired employee affective responses or behaviors, in turn diminishing the effectiveness of paternalism.

Additionally, leaders should also attach greater importance to the satisfaction of subordinates’ basic psychological needs. On the one hand, the fulfillment of these needs may promote positive employee outcomes, such as job satisfaction, organizational commitment, creativity, and organizational citizenship behaviors. On the other hand, when subordinates’ psychological needs are satisfied, they are more likely to develop a better interpersonal relationship with their supervisor, which may further strengthen the effectiveness of the supervisor’s leadership. Conversely, when subordinates’ needs are thwarted, they will not only exhibit negative outcomes, such as a stronger intention to quit and more defensive behaviors but may also develop a worse relationship with their supervisor, making it harder for the supervisor to change the situation through their leadership.

### Limitations and Directions for Future Research

The present study involves several limitations. First, our study was conducted in a school setting in China. The longstanding hierarchical administrative structure in which teachers are accustomed to the power distance may have affected the results, particularly the analysis of autonomy needs. Future studies should be carried out in more diverse contexts, and it may be valuable to compare findings across various cultural and professional backgrounds to determine whether the motivational mechanisms of trust are universally effective.

Second, because data collection was conducted at a single time-point through a questionnaire survey, we are not able to draw conclusions regarding casual relationships between our research variables. Future studies could use experimental designs to further examine the casual connections among these variables or collect longitudinal data to offer more explicit insights into these relationships.

Third, our measurement of basic psychological needs satisfaction contains several reverse items. However, Brien et al. (2012) suggested that the positive statement of items often captures more accurate information than negative items, providing a new measurement of basic psychological needs satisfaction in which all items are positively stated. Brien et al. (2012) further suggested that the separation and independent examination of need satisfaction and need frustration is a promising approach. Therefore, future research could expand on the current study by including the concept of need frustration in the model and compare their mediating roles in the facilitation and reduction of trust.

Finally, social exchange theorists have suggested that the sense of indebtedness, the gain-and-loss of resource exchanges, and the trustworthiness of ability, benevolence, and integrity are key antecedents of trust. Hence, instead of proposing a direct effect of leadership on trust, future studies could incorporate these factors as mediators to further investigate the social exchange processes that help to enhance subordinates’ decisions regarding TS.

## Conclusion

The present study sought to inform the debate between the direct instrumental perspective and the SDT-based mediating motivational perspective regarding the facilitation of trust by examining a model in which both paths are hypothesized. Our empirical results revealed that paternalistic leadership had both significant direct and indirect effects on trust, which indicates that both perspectives have explanatory power in the understanding of trust. Moreover, the greater impact of direct effects suggests that social exchange theory and other instrumental-oriented perspectives on trust may still be useful, whereas the smaller indirect effects suggest that SDT may also contribute to the understanding of close interpersonal relationships to some extent. Although trust is a central factor determining the effectiveness of leadership and organizational management, it is not clearly understood and requires further investigation. Future studies may gain more comprehensive and precise insight by exploring more mediating mechanisms regarding trust, to provide a broader and more detailed perspective.

## Data Availability Statement

The datasets presented in this article are not readily available because original data contains personal information of the research participants. Requests to access the datasets should be directed to YC, caiyonghong@bnu.edu.cn.

## Author Contributions

RT: formal analysis, data curation, writing—original draft, and visualization. YC: conceptualization, methodology, investigation, writing—original draft, project administration, and funding acquisition. HZ: data curation. All authors contributed to the article and approved the submitted version.

## Conflict of Interest

The authors declare that the research was conducted in the absence of any commercial or financial relationships that could be construed as a potential conflict of interest.

## Publisher’s Note

All claims expressed in this article are solely those of the authors and do not necessarily represent those of their affiliated organizations, or those of the publisher, the editors and the reviewers. Any product that may be evaluated in this article, or claim that may be made by its manufacturer, is not guaranteed or endorsed by the publisher.

## References

[B1] Baer M. van der Werff L. Colquitt J. A. Rodell J. B. Zipay K. Buckley F. (2018). Trusting the “look and feel”: situational normality, situational aesthetics, and the perceived trustworthiness of organizations. *Acad. Manag. J.* 61 1718–1740. 10.5465/amj.2016.0248

[B2] Balaguer I. González L. Fabra P. Castillo I. Mercé J. Duda J. L. (2012). Coaches’ interpersonal style, basic psychological needs and the well- and ill-being of young soccer players: a longitudinal analysis. *J. Sports Sci.* 30 1619–1629. 10.1080/02640414.2012.731517 23062028

[B3] Blais M. R. Sabourin S. Boucher C. Vallerand R. (1990). Toward a motivational model of couple happiness. *J. Pers. Soc. Psychol.* 59 1021–1031. 10.1037/0022-3514.59.5.1021

[B4] Blau P. M. (1964). *Exchange and Power in Social Life.* New York, NY: Wiley.

[B5] Brien M. Forest J. Mageau G. A. Boudrias J. Desrumaux P. Brunet L. (2012). The basic psychological needs at work scale: measurement invariance between Canada and France. *Appl. Psychol. Health Well Being* 4 167–187. 10.1111/j.1758-0854.2012.01067.x 26286976

[B6] Byrne B. M. (2001). *Structural Equation Modelling with AMOS: Basic Concepts, Applications, and Programming.* Mahwah, NJ: Lawrence Erlbaum Associates.

[B7] Chen B. W. Vansteenkiste M. Beyers W. Boone L. Deci E. L. van der Kaap-Deeder J. (2015). Basic psychological need satisfaction, need frustration, and need strength across four cultures. *Motiv. Emot.* 39 216–236. 10.1007/s11031-014-9450-1

[B8] Chen X. P. Eberly M. B. Chiang T. J. Farh J. L. Cheng B. S. (2014). Affective trust in Chinese leaders: linking paternalistic leadership to employee performance. *J. Manag.* 40 796–819. 10.1177/0149206311410604

[B9] Cheng B. S. Chou L. F. Wu T. Y. Huang M. P. Farh J. L. (2004). Paternalistic leadership and subordinate responses: establishing a leadership model in Chinese organizations. *Asian J. Soc. Psychol.* 7 89–117. 10.1111/j.1467-839x.2004.00137.x

[B10] Chong J. X. Y. Gagné M. (2019). “Self-determination theory for work motivation,” in *Oxford Bibliographies in Management*, ed. Griffin R. (New York, NY: Oxford University Press).

[B11] Deci E. L. Connell J. P. Ryan R. M. (1989). Self-determination in a work organization. *J. Appl. Psychol.* 74 580–590.

[B12] Deci E. L. La Guardia J. G. Moller A. C. Scheiner M. J. Ryan R. M. (2006). On the benefits of giving as well as receiving autonomy support: mutuality in close friendships. *Pers. Soc. Psychol. Bull.* 32 313–327. 10.1177/0146167205282148 16455859

[B13] Deci E. L. Olafsen A. H. Ryan R. M. (2017). Self-determination theory in work organizations: the state of a science. *Annu. Rev. Organ. Psychol. Organ. Behav.* 4 19–43.

[B14] Deci E. L. Ryan R. M. (2008). Facilitating optimal motivation and psychological well-being across life’s domains. *Can. Psychol.* 49 14–23. 10.1037/0708-5591.49.1.14

[B15] Deci E. L. Ryan R. M. (2014). “Autonomy and need satisfaction in close relationships: relationships motivation theory,” in *Human Motivation and Interpersonal Relationships: Theory, Research, and Applications*, ed. Weinstein N. (London: Springer), 53–76. 10.1007/978-94-017-8542-6_3

[B16] Deci E. L. Ryan R. M. Gagné M. Leone D. R. Usunov J. Kornazheva B. P. (2001). Need satisfaction, motivation, and well-being in the work organizations of a former Eastern Bloc country: a cross-cultural study of self-determination. *Pers. Soc. Psychol. Bull.* 27 930–942. 10.1177/0146167201278002

[B17] Dedahanov A. T. Bozorov F. Sung S. (2019). Paternalistic leadership and innovative behavior: psychological empowerment as a mediator. *Sustainability* 11 1–14.

[B18] Dirks K. T. Ferrin D. L. (2001). The role of trust in organizational settings. *Organ. Sci.* 12 450–467. 10.1287/orsc.12.4.450.10640 19642375

[B19] Eakman A. M. (2014). A prospective longitudinal study testing relationships between meaningful activities, basic psychological needs fulfillment, and meaning in life. *OTJR Occup. Particip. Health* 34 93–105. 10.3928/15394492-20140211-01 24649934

[B20] Farh J. L. Cheng B. S. (2000). “A cultural analysis of paternalistic leadership in Chinese organizations,” in *Management and Organizations in the Chinese Context*, eds Li J. T. Tsui A. S. Weldon E. (London: Macmillan), 84–127. 10.1057/9780230511590_5

[B21] Fulmer C. A. Gelfand M. J. (2012). At what level (and in whom) we trust: trust across multiple organizational levels. *J. Manag.* 38 1167–1230. 10.1177/0149206312439327

[B22] Ip P. K. (2009). Is Confucianism good for business ethics in China? *J. Bus. Ethics* 88 463–476. 10.1007/s10551-009-0120-2

[B23] Knee C. R. Lonsbary C. Canevello A. Patrick H. (2005). Self-determination and conflict in romantic relationships. *J. Pers. Soc. Psychol.* 89 997–1009. 10.1037/0022-3514.89.6.997 16393030

[B24] Kramer R. M. (1999). Trust and distrust in organizations: emerging perspectives, enduring questions. *Annu. Rev. Psychol.* 50 569–598. 10.1146/annurev.psych.50.1.569 15012464

[B25] La Guardia J. G. Ryan R. M. Couchman C. E. Deci E. L. (2000). Within-person variation in security of attachment: a self-determination theory perspective on attachment, need fulfillment, and well-being. *J. Pers. Soc. Psychol.* 79 367–384. 10.1037/0022-3514.79.3.367 10981840

[B26] Legood A. van der Werff L. Lee A. Den Hartog D. (2021). A meta-analysis of the role of trust in the leadership-performance relationship. *Eur. J. Work Organ. Psychol.* 30 1–22. 10.1080/1359432x.2020.1819241

[B27] Little T. D. (1997). Mean and covariance structures (MACS), analyses of cross-cultural data: practical and theoretical issues. *Multivariate Behav. Res.* 32 53–76. 10.1207/s15327906mbr3201_3 26751106

[B28] MacKinnon D. P. Lockwood C. M. Hoffman J. M. West S. G. Sheets V. (2002). A comparison of methods to test mediation and other intervening variables effects. *Psychol. Methods* 7 83–104. 10.1037/1082-989x.7.1.83 11928892 PMC2819363

[B29] Mascall B. Leithwood K. Strauss T. Sacks R. (2009). “The relationship between distributed leadership and teachers’ academic optimism,” in *Distributed Leadership: Different Perspectives*, ed. Harris A. (Berlin: Springer Netherlands).

[B30] Maslow A. H. (1943). A theory of human motivation. *Psychol. Rev.* 50 370–396.

[B31] Mayer R. C. Davis J. H. Schoorman F. D. (1995). An integrative model of organizational trust. *Acad. Manag. Rev.* 20 709–734. 10.5465/amr.1995.9508080335

[B32] McAllister D. J. (1995). Affect-and cognition-based trust as foundations for interpersonal cooperation in organizations. *Acad. Manag. J.* 38 24–59. 10.5465/256727 256727

[B33] Mishra M. Ghosh K. (2020). Supervisor monitoring and subordinate work attitudes: a need satisfaction and supervisory support perspective. *Leadersh. Organ. Dev. J.* 41 1089–1105. 10.1108/lodj-05-2019-0204

[B34] Nienaber A. Romeike P. D. Searle R. Schwe G. (2014). A qualitative meta-analysis of trust in supervisor-subordinate relationships. *J. Manage. Psychol.* 30 507–534.

[B35] Patrick H. Knee C. R. Canevello A. Lonsbary C. (2007). The role of need fulfillment in relationship functioning and well-being: a self-determination theory perspective. *J. Pers. Soc. Psychol.* 92 434–457. 10.1037/0022-3514.92.3.434 17352602

[B36] Pellegrini E. K. Scandura T. A. (2008). Paternalistic leadership: a review and agenda for future research. *J. Manag.* 34 566–593. 10.1177/0149206308316063

[B37] Podsakoff P. M. MacKenzie S. B. Lee J. Y. Podsakoff N. P. (2003). Common method biases in behavioral research: a critical review of the literature and recommended remedies. *J. Appl. Psychol.* 88 879–903. 10.1037/0021-9010.88.5.879 14516251

[B38] Podsakoff P. M. MacKenzie S. B. Podsakoff N. P. (2012). Sources of method bias in social science research and recommendations on how to control it. *Annu. Rev. Psychol.* 63 539–569. 10.1146/annurev-psych-120710-100452 21838546

[B39] Rempel J. K. Holmes J. G. Zanna M. D. (1985). Trust in close relationships. *J. Pers. Soc. Psychol.* 49 95–112.

[B40] Rhoades L. Eisenberger R. (2002). Perceived organizational support: a review of the literature. *J. Appl. Psychol.* 87 698–714. 12184574 10.1037/0021-9010.87.4.698

[B41] Rousseau D. M. Sitkin S. B. Burt R. S. Camerer C. (1998). Not so different after all: a cross-discipline view of trust. *Acad. Manag. Rev.* 23 393–404. 10.5465/amr.1998.926617

[B42] Ryan R. M. (2019). “Inside the black box,” in *The Oxford Handbook of Human Motivation*, ed. Ryan R. M. (New York, NY: Oxford University Press), 1–13. 10.1093/oxfordhb/9780190666453.013.1

[B43] Ryan R. M. Ryan W. S. Di Domenico S. I. Deci E. L. (2019). “The nature and the conditions of human autonomy and flourishing: self-determination theory and basic psychological needs,” in *The Oxford Handbook of Human Motivation*, ed. Ryan R. M. (New York, NY: Oxford University Press), 214–276.

[B44] van den Broeck A. Ferris D. L. Chang C. H. Rosen C. C. (2016). A review of self-determination theory’s basic psychological needs at work. *J. Manag.* 42 1195–1229. 10.1177/0149206316632058

[B45] van der Werff L. Legood A. Buckley F. Weibel A. de Cremer D. (2019). Trust motivation: the self-regulatory processes underlying trust decisions. *Organ. Psychol. Rev.* 9 99–123. 10.1177/2041386619873616

[B46] Weber M. (1968). “The types of legitimate domination,” in *Economy and Society*, eds Roth G. Wittich C. (New York, NY: Bedminster), 212–216.

[B47] Wild T. C. Enzle M. E. Nix G. Deci E. L. (1997). Perceiving others as intrinsically or extrinsically motivated: effects on expectancy formation and task engagement. *Pers. Soc. Psychol. Bull.* 23 837–848. 10.1177/0146167297238005

[B48] Williams G. C. Cox E. M. Hedberg V. Deci E. L. (2000). Extrinsic life goals and health risk behaviors in adolescents. *J. Appl. Soc. Psychol.* 30 1756–1771. 10.1111/j.1559-1816.2000.tb02466.x

[B49] Wu M. (2012). Moral leadership and work performance: testing the mediating and interaction effects in China. *Chin. Manag. Stud.* 6 284–299. 10.1108/17506141211236721

[B50] Wu M. Huang X. Li C. Liu W. (2012). Perceived interactional justice and trust-in-supervisor as mediators for paternalistic leadership. *Manag. Organ. Rev.* 8 97–121. 10.1111/j.1740-8784.2011.00283.x

[B51] Yu C. F. Li X. Zhang W. (2015). Predicting adolescent problematic online game use from teacher autonomy support, basic psychological needs satisfaction, and school engagement: a 2-year longitudinal study. *Cyberpsychol. Behav. Soc. Netw.* 18 228–233. 10.1089/cyber.2014.0385 25803769

